# An integrative approach reveals a new species of flightless leaf beetle (Chrysomelidae: *Suinzona*) from South Korea

**DOI:** 10.1038/s41598-021-88011-2

**Published:** 2021-04-21

**Authors:** Hee-Wook Cho, Sang Ki Kim

**Affiliations:** Department of Zoology, Nakdonggang National Institute of Biological Resources, Sangju, 37242 South Korea

**Keywords:** Ecology, Evolution, Molecular biology, Zoology

## Abstract

The leaf beetle genus *Suinzona,* consisting of over 20 species, is mainly known for species from Southwest China, and its closely related genus *Potaninia,* with only two species, is widely distributed in South China and upper Southeast Asia. Despite recent morphological studies, the status of these taxa has long been controversial. Here, we investigated the taxonomic status and phylogenetic relationships of the genera *Suinzona* and *Potaninia* within Chrysomelinae using molecular, morphological and ecological data. Molecular phylogenetic analysis supported that they should be regarded as distinct genera, which is consistent with morphological evidence, e.g., well-developed/reduced hind wings. Based on combined evidence from examination of larval and adult morphology, host plants and mitochondrial genomes, we demonstrate that *P*. *cyrtonoides* should be placed in the genus *Suinzona* and that specimens from South Korea represent a new species. *Suinzona borowieci* sp. nov., occurring in narrow strips of habitat, shows high levels of genetic divergence and distantly related host plants between populations. The population differentiation seems to be correlated with its non-functional wings causing reduced dispersal ability and genetic isolation. Several populations have declined dramatically over the last few decades due to loss of habitat and thus are in need of protection as conservation units.

## Introduction

The subfamily Chrysomelinae is one of the most diverse groups of Chrysomelidae, with approximately 3000 species and subspecies in 130 genera occurring in most parts of the world^1^. Adults of Chrysomelinae are typically medium sized, semi-ovoid in shape, brightly coloured or dull, and dorsally glabrous, while the larvae are elongated and often dorsally convex, with numerous sclerotized setose tubercles and coloured integuments. Both larvae and adults of almost all species of Chrysomelinae feed externally on leaves of the same host plant, generally with a narrow host range at the generic and species levels^2^.

*Suinzona* is a small genus of wingless chrysomelid beetles endemic to China, where its species are restricted to Sichuan and Yunnan provinces, especially in the Hengduan Mountains region^3^. The genus was established by Chen^4^ for a single species, *Suinzona laboissierei* Chen, 1931, from Sichuan and was distinguished from the presumedly closely related genus *Potaninia* Weise, 1889, by the absence of pronotal trichobothria, invisible lateral margins of the pronotum from above, elytra strongly narrowing behind, reduced hind wings, and extremely short metaventrite. After 30 years, Chen^5^ described a second species, *Suinzona monticola,* from Sichuan, which was the last description of a new *Suinzona* species before the 2000s. However, the status of *Suinzona* has been controversial from the beginning. Chen^6^ once synonymized *Suinzona* with *Potaninia* without comment in his catalogue. While some authors have treated *Suinzona* as a distinct genus^7–9^, others have treated it as a synonym of *Potaninia*^10–12^ or as a subgenus of *Potaninia*^13^. Recently, Ge et al.^3^presented a review and cladistic analysis of the closely related genera *Suinzona* Chen, *Potaninia* Weise and *Taipinus* Lopatin. In their revision, *Suinzona* was considered a distinct genus with an emended diagnosis, 15 new species were described with a key to the species, and six described species were transferred to *Suinzona*. A total of 23 species are currently known, of which 21 occur in Sichuan and two in Yunnan. The genus *Potaninia* includes only two species, *P*. *assamensis* (Baly, 1879), found in China, India, Laos, Myanmar, Thailand and Vietnam, and *P*. *cyrtonoides* (Jacoby, 1885), found in Japan and South Korea. Two characters, i.e., well-developed hind wings and the presence of humeral calli, were used in the key to genera of Chinese Chrysomelinae by Yang et al.^14^, distinguishing them from *Suinzona*. Examination of the latter character indicates that ‘the presence of humeral calli of elytra’ is common in winged beetles, whereas this character may be reduced to absent in apterous or reduced-winged beetles^15^.

During the examination of type materials of *P. cyrtonoides* collected in Japan, the first author found that all of them had reduced hind wings, which is the key diagnostic character of the genus *Suinzona*. After examining a large amount of material from museum collections and recent field collection surveys in South Korea and Japan, we observed ‘reduced wings (= steno- and brachypterous)’, which was wrongly interpreted as ‘present (= well-developed, macropterous)’ by Ge et al.^3^, with the absence of humeral calli in all specimens. Molecular analysis also supports its generic placement, i.e., *Suinzona cyrtonoides* (Jacoby, 1885) comb. nov. Moreover, we found that specimens from South Korea, previously identified as *P*. *cyrtonoides*, represent a new species, *Suinzona borowieci* sp. nov. The two above species share a quadrifid tip of the flagellum of the aedeagus, which is a remarkable and unique feature within *Suinzona*. The present paper aims to describe a new species of the genus *Suinzona* with the reassignment of *P. cyrtonoides* to *Suinzona* based on the morphological characters of larvae and adults, host plant association and the mitochondrial genome.

## Results

### Description of ***Suinzona borowieci*** sp. nov. (Figs. 1, 2 and 3)

**Figure 1:**
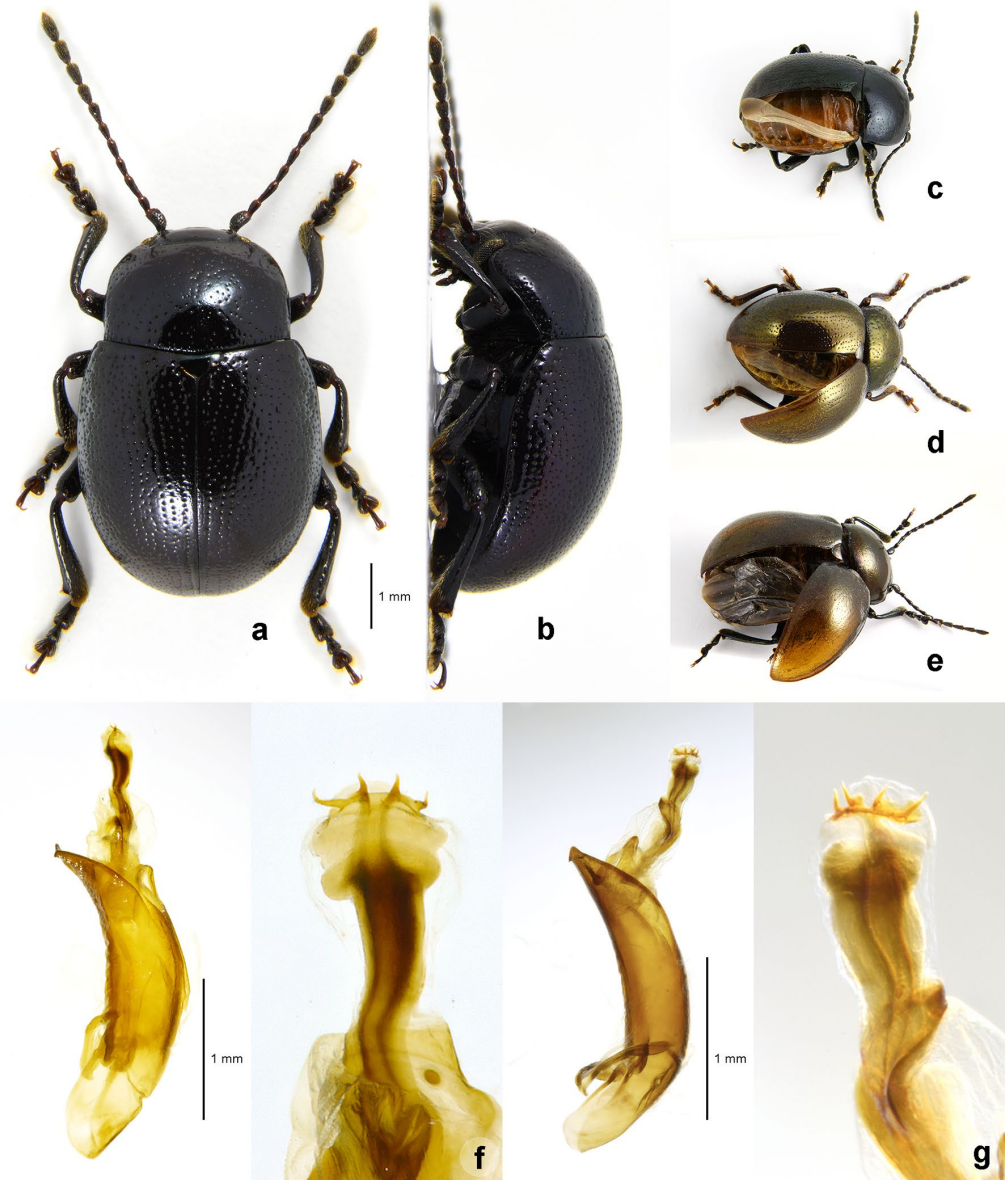
Morphology of *Suinzona borowieci* sp. nov. and related species: (a,b) Holotype of *S. borowieci* sp. nov. (a) Dorsal habitus, (b) lateral habitus; (c–e) exposed hind wing, (c) *S. borowieci* sp. nov., (d) *S. cyrtonoides*, (e) *Potaninia assamensis*; (f–g) aedeagus with everted internal sac (left) and flagellum (right); (f) *S. borowieci* sp. nov., (g) *S*. *cyrtonoides*.

**Figure 2:**
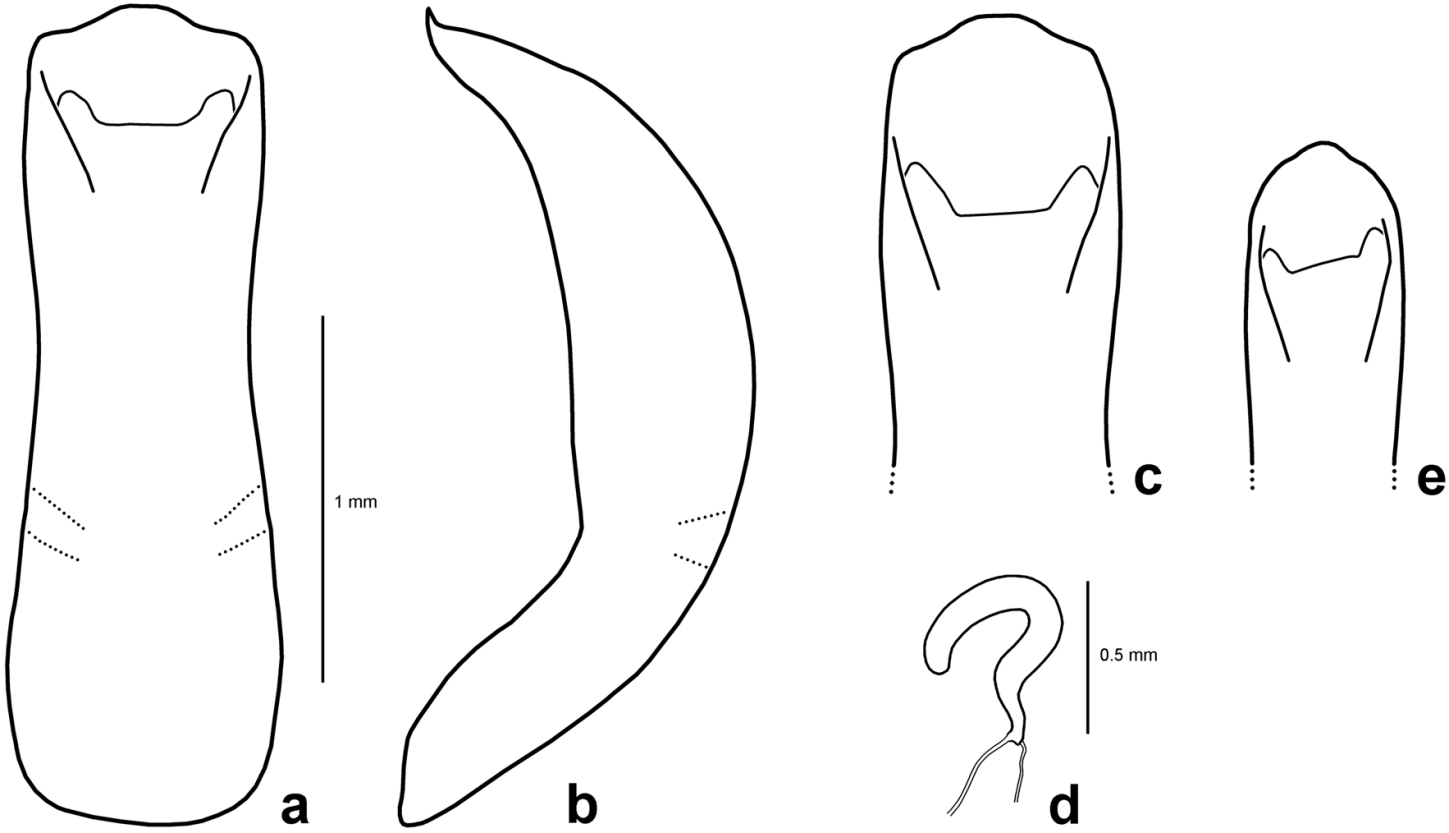
Genitalia of *Suinzona borowieci* sp. nov. and related species: (a–d) *S*. *borowieci* sp. nov. (a) Aedeagus, dorsal view; (b) aedeagus, lateral view; (c) aedeagus, apical view; (d) spermatheca. (e) Aedeagus of *Suinzona cyrtonoides*, apical view.

**Figure 3:**
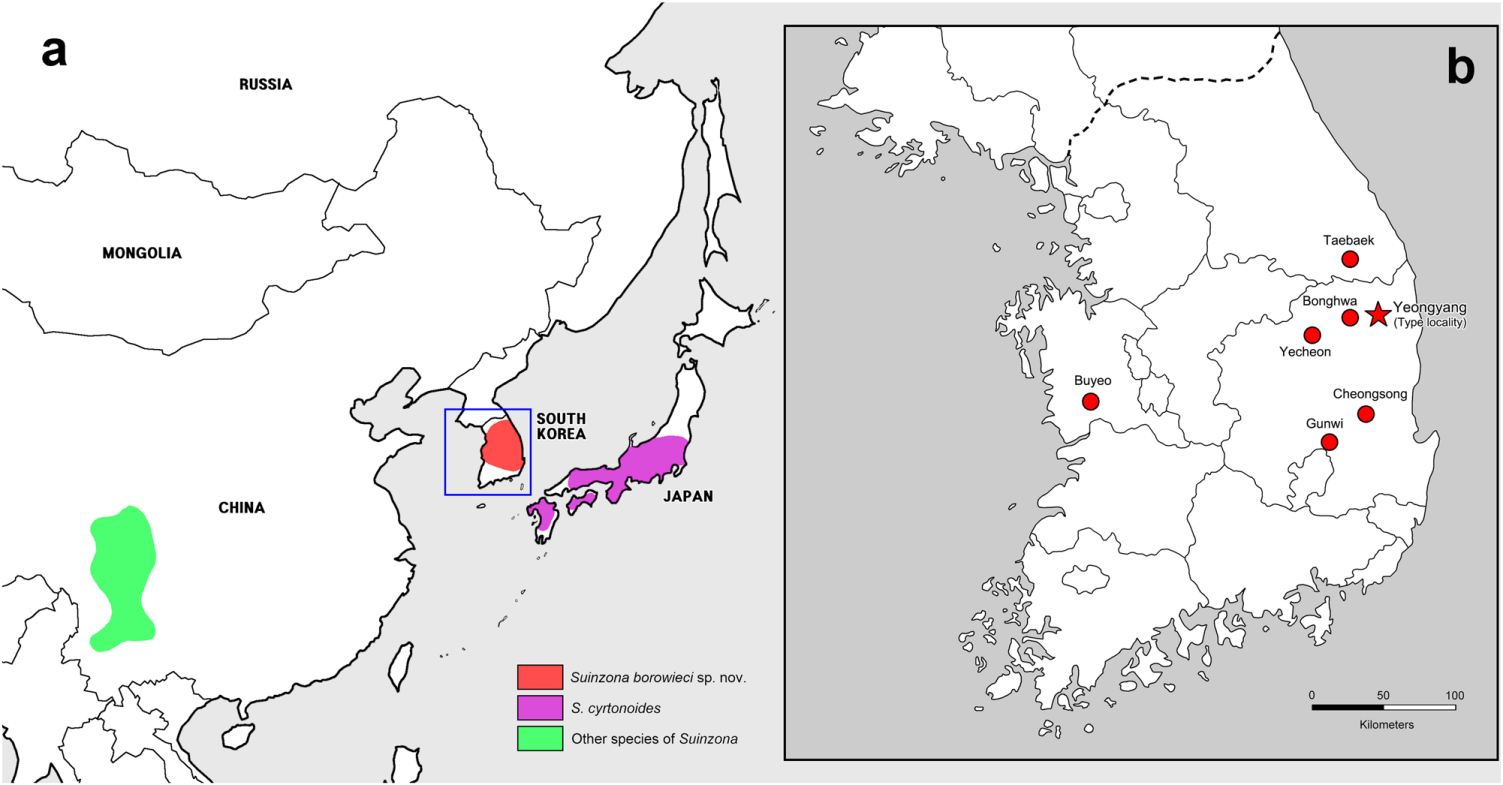
Distribution map of *Suinzona* and sampling sites: (a) Distribution of *Suinzona* species in China, South Korea and Japan, (b) type locality and collection sites of *Suinzona borowieci* sp. nov. in South Korea. Records of distribution are taken from Ge et al.^3^, Suzuki et al.^21^ and the results of this work. The map is redrawn and modified from National Geographic Information Institute of Korea (https://www.ngii.go.kr).

Family Chrysomelidae Latreille, 1802

Subfamily Chrysomelinae Latreille, 1802

Genus *Suinzona* Chen, 1931

#### Type locality

South Korea: Gyeongbuk Province, Yeongyang County, Irwolsan Mountain, 36° 48′ 30.42" N, 129° 5′ 23.56" E, ca. 1135 m.

#### Type material

**Holotype:** male (NMPC), South Korea: Gyeongbuk Prov., Yeongyang, Mt. Irwolsan, 36° 48′ 30.42" N, 129° 5′ 23.56" E, ca. 1135 m, 12.VI.2011, H.W. Cho // HOLOTYPUS *Suinzona borowieci* sp. n. Cho & Kim 2020. **Paratype**: SOUTH KOREA – **Gyeongbuk Prov**.: 1 female (NMPC), same data as holotype plus PARATYPUS *Suinzona borowieci* sp. n. Cho & Kim 2020; 1 female (HCC), same data as holotype except 31.VII.2004; 1 female (HCC), same data as holotype except 31.VII.2004; 4 males 2 females (HCC), same data as holotype except 22.V.2009; 8 males 2 females (HCC), same data as holotype except 25.VI.2010; 4 males 2 females (HCC), same data as holotype except 10.VI.2017; 1 male 1 female (HCC), same data as holotype except 17.VI.2017; 1 male (HCC), same data as holotype except 36° 48′ 11.74" N, 129° 6′ 10.01" E, ca. 1190 m, 17.V.2020; 3 males 1 female (HCC), same data as holotype except 7.VI.2020; 2 males (KNAE), Yeongyang, Irwol-myeon, Mt. Irwolsan, 7.VI.2014, J.K. Park // I14_KNAE483613 // I14_KNAE483649; 1 male 1 female (HCC), Bongwha, Myeongho-myeon, Bukgok-ri, Mt. Cheongnyangsan, 36° 47′ 47" N, 128° 54′ 30" E, 21–22.V.2015, J.S. Lee; 1 female (HCC), Daegu, Dong-gu, Mt. Palgongsan, 21.V.1998; 2 males 1 female (HCC), Gunwi, Bugye-myeon, Dongsan-ri, Mt. Palgongsan, 9.V.2009, S.S. Jung; 1 male 1 female (HCC), Yecheon, Bomun-myeon, Urae-ri, Mt. Hakgasan, 26.V.2010, Y.J. You; 1 male (HCC), Yecheon, Bomun-myeon, Mt. Hakgasan, 36° 40′ 32.16" N, 128° 35′ 38.24" E, ca. 330 m, 3.VI.2020, H.W. Cho; 1 female (HCC), Cheongsong, Hyeonseo-myeon, Galcheon-ri, 26.V.2004, H.W. Cho; **Gangwon Prov**.: 2 females (HCC), Taebaek, Hwangji-dong, Mt. Hambaeksan, 37° 9′ 53.22" N, 128° 55′ 1.35" E, ca. 1470 m, 6.VI.2005, H.W. Cho; 2 males 3 females (HCC), same data as preceding one except 6.VI.2006; 1 female (HCC), same data as preceding one except 29.V.2009; 1 female (HCC), same data as preceding one except 10.VI.2017; 1 female (HCC), same data as preceding one except 5.VI.2020; **Chungnam Prov**.: 1 male (HCC), Buyeo, Gyuam-myeon, Sumok-ri, 1–15.VI.2005, J.W. Lee.

#### Other material

Six mature larvae (HCC), same data as holotype except 29.VI.2017; 5 mature larvae (HCC), Gangwon Prov., Taebaek, Hwangji-dong, Mt. Hambaeksan, 19.VI.2006, H.W. Cho; 8 mature larvae (HCC), Gyeongbuk Prov., Yecheon, Bomun-myeon, Mt. Hakgasan, 31.V.2020, H.W. Cho; 7 mature larvae (HCC), same data as preceding one except 3.VI.2020.

#### Diagnosis

*Suinzona borowieci *sp. nov. is almost identical to *S*. *cyrtonoides* in the shape of the flagellum of the aedeagus. However, it can be distinguished by its larger body size (5.5–7.0 mm vs. 4.8–6.0 mm), denser punctures on elytra (less dense punctures in *S*. *cyrtonoides*), larger and broader aedeagus with the distal tips of the flagellum quadrifurcated and slightly curved, arising from two sclerotized tubes (with a smaller and narrower aedeagus with distal tips of the flagellum quadrifurcated and almost straight, arising from a sclerotized tube in *S*. *cyrtonoides*).

#### Description

Measurements in mm (n = 5): length of body: 5.50–7.00 (mean 6.18); width of body: 3.50–4.50 (mean 3.97); height of body: 2.60–3.40 (mean 2.94); width of head: 1.65–1.95 (mean 1.81); interocular distance: 1.15–1.50 (mean 1.33); width of apex of pronotum: 1.90–2.20 (mean 2.02); width of base of pronotum: 2.70–3.25 (mean 2.94); length of pronotum along midline: 1.75–2.05 (mean 1.90); length of elytra along suture: 3.75–5.20 (mean 4.41). Body: oval and strongly convex (Fig. 1a,b). Body dark bluish-black with weak metallic lustre, rarely with a dark brass dorsum. Antenna, mouthparts and tarsus partially dark reddish-brown. ***Head.*** Vertex weakly convex, covered with sparse punctures, becoming coarser and denser towards sides, with convex area above antennal insertion. Eyes strongly transverse-oblong and protuberant. Frontal suture V-shaped, forming obtuse angle, arms bent at middle, reaching anterior margin. Frons flat, strongly depressed at anterior margin, covered with dense punctures. Clypeus narrow and trapezoidal. Anterior margin of labrum weakly concave. Mandibles with 2 blunt apical teeth and dense punctures bearing setae on outer side. Maxillary palp 4-segmented with apical palpomere fusiform, truncate apically. Antennae in males much longer than half the length of the body; antennomere 1 robust; antennomere 2 shorter than 3; antennomere 3 longer than 4; antennomeres 7–10 each moderately widened, much longer than wide; antennomere 11 longest, approximately 2.4 times as long as wide. Antennae in females less than half the length of the body. ***Pronotum.*** 1.50–1.63 times as wide as long. Lateral sides widest at or near base, roundly narrowed anteriorly, anterior angles strongly produced. Anterior and lateral margins bordered, lateral margins barely visible in dorsal view. Trichobothria present on posterior angles. Disc glabrous, covered with moderately dense punctures, becoming coarser along basal margin; interspaces covered with fine and moderately dense punctures. Scutellum much wider than long, widely rounded apically, with a few fine punctures. ***Elytra.*** 1.07–1.16 times as long as wide. Lateral sides widest near middle, roundly narrowed posteriorly. Humeral calli not developed. Disc glabrous and finely rugose, covered with rather irregular punctures arranged in longitudinal rows near suture and lateral margin, more irregular in median region; interspaces covered with fine and sparse punctures. Epipleura wholly visible in lateral view. Hind wings steno- and brachypterous (Fig. 1c). ***Venter.*** Hypomera weakly rugose, with a few punctures near anterolateral corners of prosternum. Prosternum covered with coarse and dense punctures bearing long setae; prosternal process broad and strongly expanded apicolaterally, closing procoxal cavities posteriorly. Metasternum covered with punctures bearing long setae, dense medially, sparse laterally. Abdominal ventrites covered with moderately dense punctures bearing long or short setae; apex of last visible abdominal ventrite deeply emarginate in males while rounded in females. ***Legs.*** Moderately robust. Tibiae simple without preapical tooth. Tarsomere 1 subequal in width to tarsomere 3 in males but distinctly narrower than tarsomere 3 in females. Tarsal claws simple. ***Genitalia.*** Aedeagus broad, lateral margins shallowly concave, with apex moderately produced and truncate in dorsal view (Fig. 2a,c); regularly curved, tapering from middle to apex, with apex pointed and slightly bent upward in lateral view (Fig. 2b); flagellum club-shaped with sharp, sclerotized and quadrifid tips (Fig. 1f). Spermatheca U-shaped, long and rounded at apex (Fig. 2d).

#### Etymology

Dedicated to the first author's mentor Prof. dr hab. Lech Borowiec (University of Wrocław, Poland), the world’s leading specialist in tortoise beetles.

#### Distribution

South Korea: Chungnam, Gangwon, Gyeongbuk, Daegu (Fig. 3a,b).

#### Remarks

The shape of the apical part of the male genitalia exhibits a certain degree of variation even within the same population. It is difficult to recognize a significant difference in the shape of the male genitalia between populations, but individuals from Yeongyang have a relatively large aedeagus. All specimens that we examined had a dark bluish-black dorsum with a weak metallic lustre, but a single specimen with a dark brass dorsum was found in Yecheon.

### Mature larva and biology of *Suinzona borowieci* sp. nov. (Figs. 4, 5 and 6)

#### Diagnosis

The fourth (last) instar larva of *S. borowieci* sp. nov. is very similar to that of *S*. *cyrtonoides* comb. nov. in body shape, colouration and tubercular pattern. However, this species can be distinguished by the 4–5 small secondary tubercles between Dae and DLai on the meso- and metathorax and more densely setose bodies (1 large tubercle between Dae and DLai on the meso- and metathorax and less densely setose body in *S*. *cyrtonoides*).

**Figure 4:**
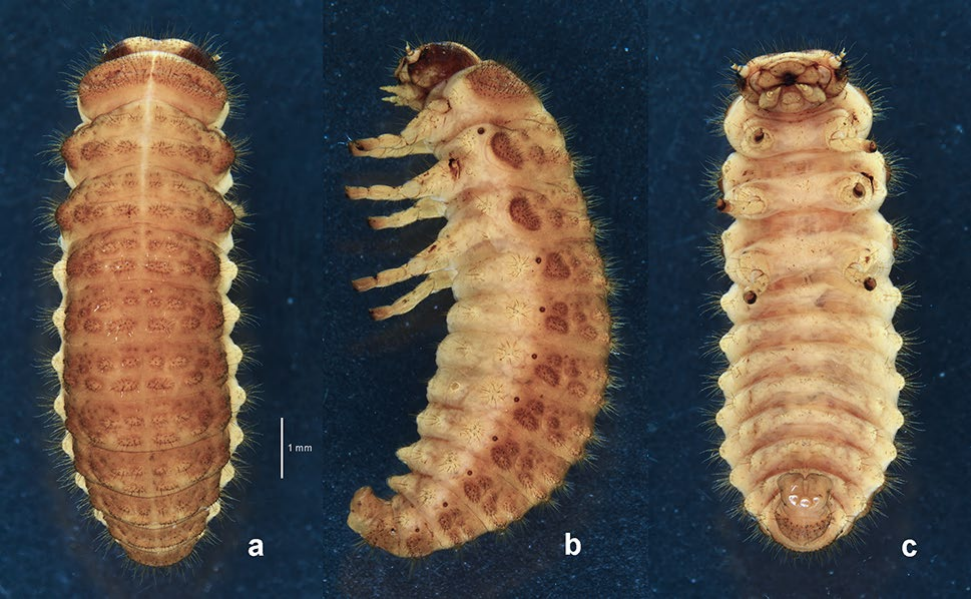
Mature larva of *Suinzona borowieci* sp. nov.: (a) Dorsal habitus, (b) lateral habitus, (c) ventral habitus.

**Figure 5:**
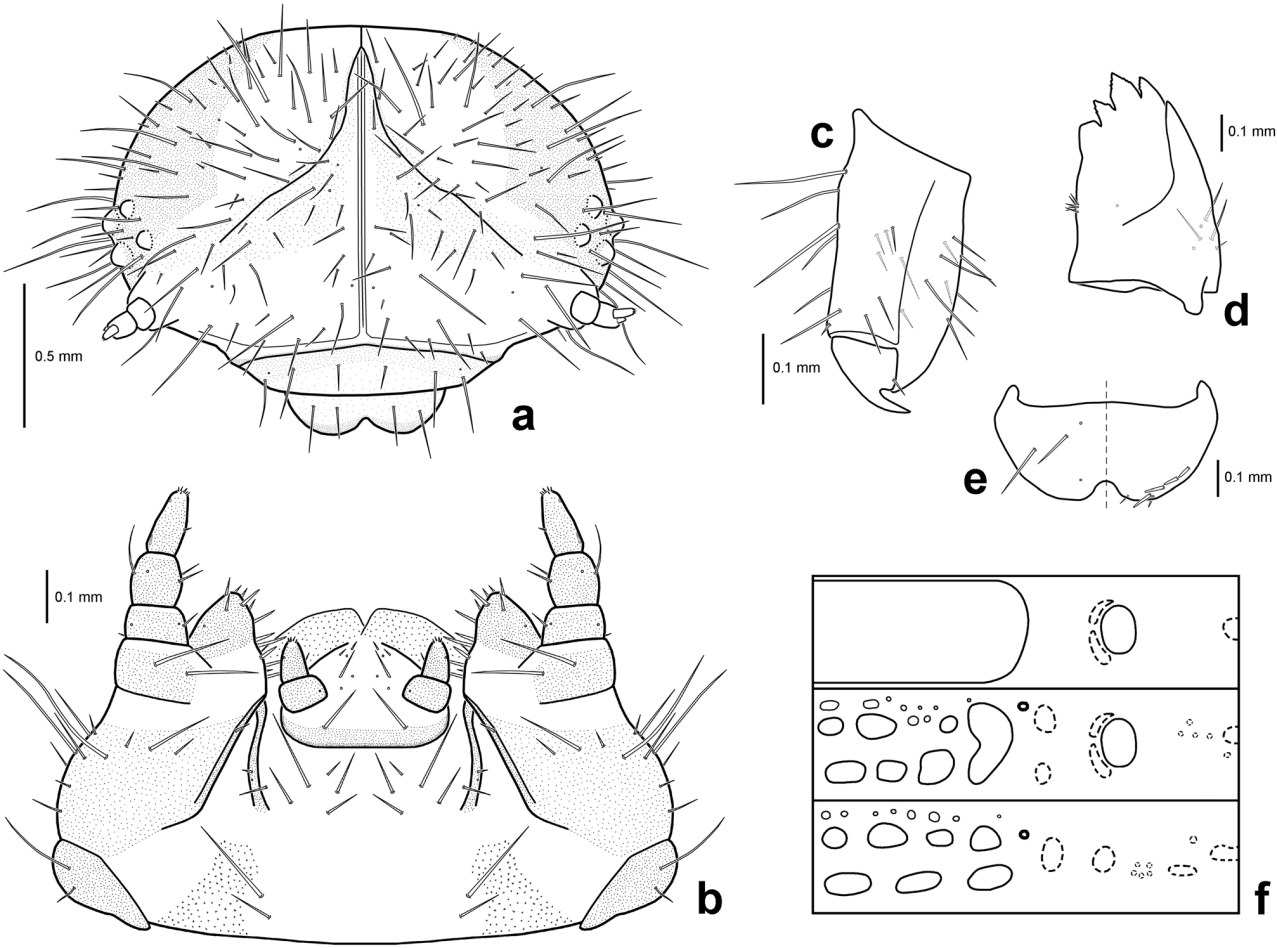
Larval morphology of *Suinzona borowieci* sp. nov.: (a) Head, (b) maxillae and labium, (c) tibiotarsus and pretarsus, (d) mandible, (e) labrum and epipharynx, (f) Schematic presentation of tubercular patterns (top: prothorax; middle: mesothorax; bottom: 2nd abdominal segment).

**Figure 6:**
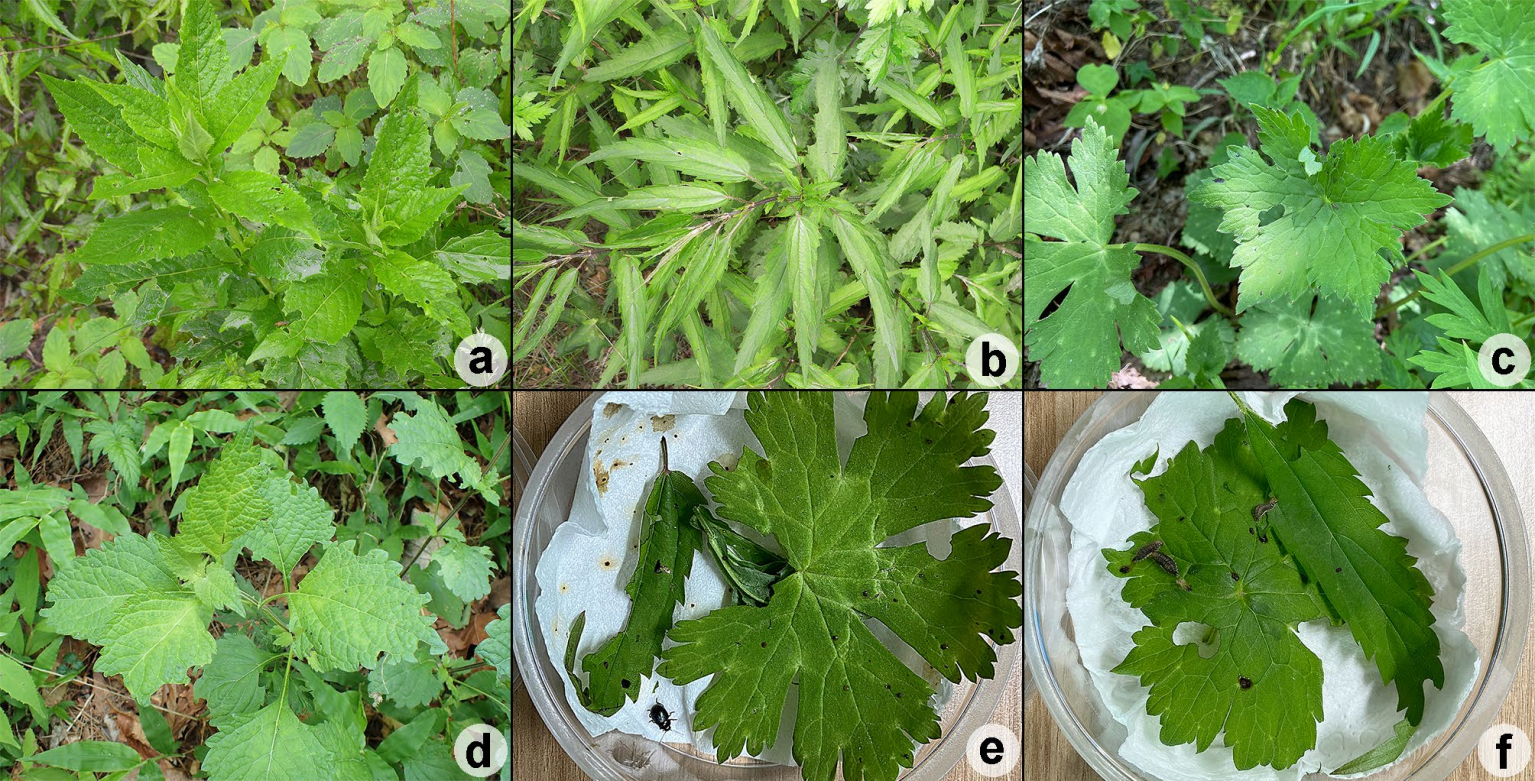
Host plants of *Suinzona borowieci* sp. nov.: (a) *Arabis pendula* L. from Yeongyang, (b) *Urtica angustifolia* Fisch. ex Hornem. from Yeongyang, (c) *Aconitum pseudolaeve* Nakai from Taebaek, (d) *Isodon inflexus* (Thunb.) Kudo from Yecheon; (e–f) *A*. *pseudolaeve* Nakai and *U*. *angustifolia* Fisch. ex Hornem. for laboratory tests (e) Adult from Yeongyang feeding on leaves, (f) larvae from Yecheon feeding on leaves.

#### Description

Body length 8.1–8.8 mm, width 2.9–3.2 mm, head width 1.75–1.80 mm (n = 3). Body elongate, rather broad, widest at abdominal segments III–IV, thence moderately narrowed posteriorly and slightly convex dorsally (Fig. 4a). Head pale yellowish-brown, densely setose, with a blackish-brown V-shaped mark along frontal arms; lateral regions of epicrania largely blackish-brown; posterior half of clypeus brown to dark brown; apex of labrum and mandibles blackish-brown. General colouration of integument yellowish-white, but dorsal integument densely covered with minute brown spinules (Fig. 4b); dorsal tubercles dark brown and ventral ones unpigmented (Fig. 4c), both densely setose; spiracles blackish-brown. Legs pale yellow with apex of tibiotarsus and pretarsus brown. Eversible glands absent. Pseudopods present on abdominal segments VI–VII. ***Head.*** Hypognathous, rounded, strongly sclerotized (Fig. 5a). Epicranium with 72–77 pairs of setae of varying length; epicranial stem distinct; frontal arms V-shaped, slightly sinuate, not extending to antennal insertions; median endocarina distinct, extending to frontoclypeal suture. Frons slightly depressed medially with 25–29 pairs of setae of varying length. Clypeus almost straight at anterior margin with 3 pairs of setae. Labrum deeply concave anteriorly with 2 pairs of setae and 2 pairs of campaniform sensilla (Fig. 5e, left); epipharynx with 6–7 pairs of setae at anterior margin (Fig. 5e, right). Mandible robust, palmate and 5-toothed, with 4–5 setae and 3 campaniform sensilla; penicillus present (Fig. 5d). Maxillary palp 3-segmented; palpomere I rectangular with 2 setae and 2 campaniform sensilla; II swollen cylindrical with 3 setae and 1 campaniform sensillum; III subconical with 1 seta, 1 digitiform sensillum and 1 campaniform sensillum on sides and a group of peg-like sensilla at the apex; palpifer well developed with 2 setae (Fig. 5b). Mala rounded with 13–14 setae and 1 campaniform sensillum; stipes distinctly longer than wide with 12–14 setae; cardo with 2–3 setae. Labial palp 2-segmented; palpomere I rectangular with 1 campaniform sensillum; II subconical with 1 seta, 1 campaniform sensillum and a group of peg-like sensilla at the apex. Hypopharynx bilobed, densely covered with minute spinules; prementum with four pairs of setae and three pairs of campaniform sensilla; postmentum basolaterally covered with minute spinules, with 8–9 pairs of setae. Six stemmata present on each side, 4 of them located above the antenna and 2 behind the antenna. Antenna 3-segmented; antenomere I wider than long with 2 campaniform sensilla; II approximately as wide as long, with a conical sensorium and 3–4 min setae; III subconical with 5–6 min setae. ***Thorax.*** Prothorax with D-DL-EP (dorsal, dorsolateral and epipleural tubercles fused together, 164–179) largest; P (pleural tubercle, 9–11) and ES-SS (eusternal and sternellar tubercles fused, 6–7) unpigmented (Fig. 5f). Meso- and metathorax with dorsal tubercles more or less arranged in 3 transverse rows; Dai (dorsal anterior interior, 6–10) on both sides separated, smaller than Dae (dorsal anterior exterior, 11–15); DLai (dorsolateral anterior interior, 4–5); Dpi (dorsal posterior interior, 12–15); Dpe (dorsal posterior exterior, 10–13) smaller than Dpi; DLpi (dorsolateral posterior interior, 17–19); DLe (dorsolateral exterior, 40–47) large; dorsal region with 8–9 secondary tubercles, 3 of them located anterior to Dai and Dae, 4–5 between Dae and DLai and 1 anterior to DLe; EPa (epipleural anterior, 17–22) larger than EPp (epipleural posterior, 8–11), both unpigmented; P (9–13), SS (1) and ES (3–4) unpigmented; sternal region with 4–5 additional setae arising from weakly sclerotized base. Mesothoracic spiracles annuliform and largest. Legs moderately long, 5-segmented; tibiotarsus with 23–25 setae; pretarsus large, strongly curved, basal tooth well developed, with 1 short seta (Fig. 5c). ***Abdomen.*** Segments I–VI with dorsal tubercles arranged in 3 transverse rows; Dai (5–8) on both sides separated, smaller than Dae (13–14); DLae (12–14) larger than DLai (7); Dpi (16–19), Dpe (15–19) and DLp (24–29) transverse, subequal in size; dorsal region with 5–10 small secondary tubercles; EP (23–27), P (12–13), PS-SS (parasternal and sternellar tubercles fused, 5–7) and ES (5–7) unpigmented; as1 (secondary tubercle on antero-exterior part of ES, 1) and as2 (secondary tubercle between P and PS, 1); sternal region with 3–4 additional setae arising from weakly sclerotized base. Segment VII with Dai and Dae fused and Dpi and Dpe fused. Segments VIII with dorsal and dorso-lateral tubercles completely fused (30–37). Segment IX with dorsal to epipleural tubercles completely fused (34–36). Segment X not visible from above, with paired pygopods. Spiracles annuliform, present on segments I–VIII.

#### Host plants

Brassicaceae: *Arabis pendula* L.; Lamiaceae: *Isodon inflexus* (Thunb.) Kudo; Ranunculaceae: *Aconitum pseudolaeve* Nakai; Urticaceae: *Urtica angustifolia* Fisch. ex Hornem.

#### Biological notes

*Suinzona borowieci* sp. nov. is univoltine. Overwintered adults appear in late May. They mate and lay 15–18 eggs per cluster on the leaves of host plants in early June. Eggs are pale yellow to yellowish-orange and hatch after 8–9 days. The larvae are solitary during the instar stages and feed on the leaves. There are four larval instars, and pupation occurs in soil. The larvae take 14–16 days to pupate and then take 7–8 days to emerge as adults. Newly emerged adults are found during July. We observed larvae or adults of this species in nearby localities (~ 62 km), feeding on *A. pendula* L. (Fig. 6a) and *U. angustifolia* Fisch. ex Hornem. (Fig. 6b) from Yeongyang (at 1135 ~ 1190 m a.s.l.), *A. pseudolaeve* Nakai (Fig. 6c) from Taebaek (at 1,470 m a.s.l.), and *I. inflexus* (Thunb.) Kudo (Fig. 6d) from Yecheon (at 330 m a.s.l.). Each population showed a preference for its natural host plant but fed on other host plants and completed its life cycle in laboratory tests (Fig. 6e,f).

### *Suinzona cyrtonoides* (Jacoby, 1885) comb. nov. (Figs. 1, 2 and 3)

#### Type locality

Japan: Kyushu, Kumamoto Prefecture, Konose.

#### Type material

**Syntypes:** 1 female (BMNH), Lectotype [mislabelled, not lectotype] // Type // DATA under card // Japan, G. Lewis, 1910–320. // *Chrysomela crytonoides* Jac. // Lectotype, *Chrysomela crytonoides* Jacoby, Designated. S. GE 2004 // *Potaninia cyrtonoides* Jacoby, Det. S. GE 2004 // *Suinzona cyrtonoides* (Jacoby, 1885) det. H.W. Cho 2014; 1 female (BMNH), Japan, G. Lewis, 1910–320. // Paralectotype // Paralectotype, *Chrysomela crytonoides* Jacoby, Designated. S. GE 2004 // *Potaninia cyrtonoides* Jacoby, Det. S. GE 2004 // *Suinzona cyrtonoides* (Jacoby, 1885) det. H.W. Cho 2014; 1 male (MCZC), Japan Lewis // 1st Jacoby Coll. // *cyrtonoides* Jac. // Type 17,474; 1 female (MCZC), Japan Lewis // 1st Jacoby Coll.

#### Other material

JAPAN – **Kyushu**: 1 male (BMNH), Yuyama 1883 // Japan, G. Lewis, 1910–320. // Paralectotype [mislabelled, not type series] // Paralectotype, *Chrysomela crytonoides* Jacoby, Designated. S. GE 2004 // *Potaninia cyrtonoides* Jacoby, Det. S. GE 2004 // *Suinzona cyrtonoides* (Jacoby, 1885) det. H.W. Cho 2014; **Honshu**: 3 males 2 females (KMNH), Nippara, Okutama, Tokyo, 5.VI.1955, Y. Tominaga; 2 males 3 females (BMNH), Mt. Mitake, Ome-shi, Tokyo, 15.VII.2005, Y. Komiya; 1 male (HSC), Chichibu, Saitama Pref., 18.VI.1984, M. Minami; 1 male (HSC), Tochigi, Sano-shi, Tanuma, 4.VI.2008, H. Ohkawa; 1 male (HSC), Gumma, Fujioka-shi, Mikabo-yama rindo, 8.VI.2009, H. Ohkawa; 1 male 2 females (HSC), same data as preceding one except 21.VII.2009; 1 male 1 female (HSC), same data as preceding one except 1.V.2010; **Shikoku**: 1 female (HSC), Tokushima, Yoshinokawa-shi, Mt. Kotsu-zan, 18.V.1987, S. Mano; 2 females (EUMJ), Tokushima, Mt. Tsurugi, 15.VII.1984, M. Miyatake; 1 male 1 female (EUMJ), Ehime: Omogo-Sibukusa, Kamiukena-gun, 5.VI.2005, Y. Satoh; 7 males (HCC), Ehime, Kamiukena, Kumakogen, Wakayama, 33° 43′ 59.4" N, 133° 08′ 06.5" E, 5.VI.2019, H.W. Cho & Y. Hiroyuki; 1 male (HSC), Ehime, Saijo-shi, Mt. Ishizuchizan, 30.V.2009, H. Suenaga; 2 males (HSC), Ehime, Saijo-shi, Nishinokawa, 16.V.2010, H. Suenaga; 1 male 1 female (HSC), Ehime, Saijo-shi, Nishinokawa, 5.VI.2010, H. Suenaga; 1 female (EUMJ), Jiyoshi-toge, Ehime Pref., 26.IV.1976, A. Oda; 1 male (EUMJ), Mt. Ishizuchi, Ehime pref., 1.VI.1975, H. Kan; 1 female (EUMJ), Iwayaji, Ehime Pref., 1.VI.1969, M. Miyatake; 1 male (EUMJ), Ehime: Yokono, 750 m alt. Yanadani-mura, 7.V.1994, M. Sakai; 1 male (EUMJ), Ehime: Yokono, 660 m alt. Yanadani-mura, 6.V.1994, M. Sakai; 1 female (EUMJ), Ehime: Yokono, 700 m alt. Yanadani-mura, 15.VII.1994, M. Sakai.

#### Distribution

Japan: Honshu, Shikoku, Kyushu (Fig. 3a).

#### Host plants

Urticaceae: *Boehmeria spicata* (Thunb.) Thunb., *Boehmeria tricuspis* (Hance) Makino.

#### Biological notes

Detailed descriptions of larvae and pupae and the life cycle have been published by Kimoto^16^ and Kimoto and Takizawa^11^. Its life cycle is similar to that of *S*. *borowieci* sp. nov., but they feed on different host plants.

#### Remarks

The apical part of the aedeagus is highly variable, narrow to broad, apex narrowly to widely rounded or weakly truncate, mainly with two weak or strong denticles on the apicolateral margin. The aedeagus of the type specimen is narrowly rounded without apicolateral denticles (Fig. 2e). However, we were not able to find an obvious tendency in the morphological variation of the aedeagus at the intrapopulation or interpopulation level. *Chrysomela cyrtonoides* Jacoby, 1885 was described from Japan. Later, it was transferred to the genus *Potaninia* by Chûjô and Kimoto^17^ and then accepted by various authors until now. However, we found that all materials of *P*. *cyrtonoides* have reduced hind wings (Fig. 1d), which are the key diagnostic features of the genus *Suinzona,* and molecular analysis also suggests its placement in *Suinzona*. Therefore, *Suinzona cyrtonoides* (Jacoby, 1885) comb. nov. is proposed. Jacoby^18^ gave ‘Konose’ as the type locality and used at least two specimens collected by G. Lewis for the description. A male specimen (BMNH) from ‘Yuyama’, designated by Ge et al.^3^ as a lectotype, did not belong to the type series of *S*. *cyrtonoides* and thus lost its lectotype status (ICZN: Article 74.2). Indeed, a female specimen (BMNH) was mislabelled as a lectotype. We were able to find four specimens collected from Japan that might belong to the type series of *S*. *cyrtonoides* in the G. Lewis collection (BMNH, MCZC), but more precise locality data were not available. Therefore, we regard them as syntypes and defer selection of a lectotype.

### Molecular phylogenetic analyses

**Figure 7:**
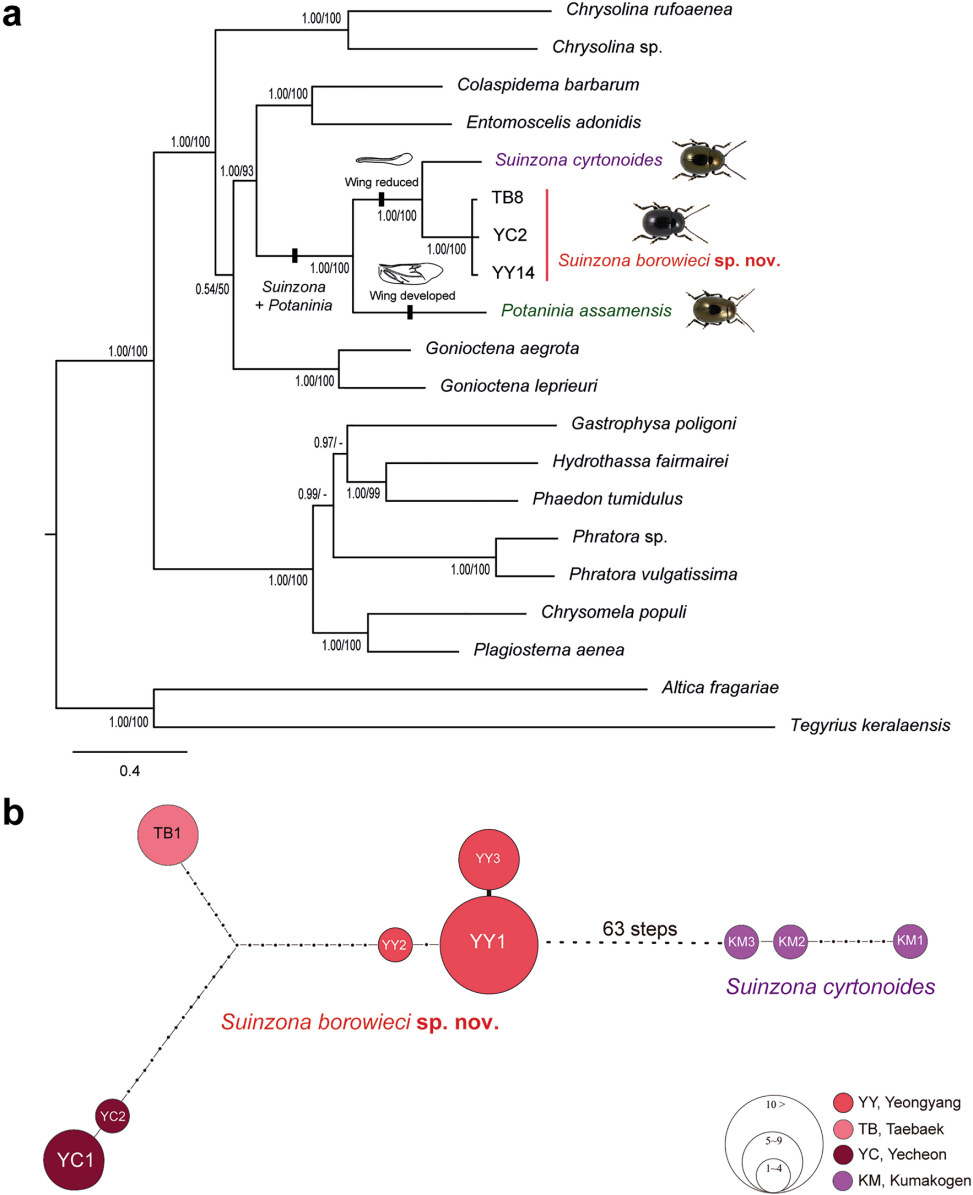
Phylogenetic tree and parsimonious network: (**a**) Bayesian consensus tree inferred from the combined mitochondrial 13 PCGs + 2 rRNA gene. Bayesian inference (left) and maximum likelihood (right) support values are shown on the branch nodes. Only the values over 70% are reported, (**b**) Parsimonious network of COI haplotypes. Circles correspond to haplotypes, the frequency and geographic origin of which are indicated by circle size. The geographical origins of the haplotypes are noted at the bottom right of the figure.

It is evident from the clarified phylogenetic inference based on mitogenomes that the genus *Suinzona* differs from the genus *Potaninia*, *S. borowieci* sp. nov. as the sister species of *S. cyrtonoides* (Fig. 7a). The phylogenetic inferences included a total of 20 mitogenomes of Chrysomelinae and outgroups of Galerucinae (Supplementary Table S1). The complete mitogenomes of the four *Suinzona* species and one *Potaninia* species (incomplete) were newly sequenced in the present study. Each mitogenome contains a typical set of mitochondrial genes (13 PCGs, 22 tRNAs and two rRNAs) and a control region. Phylogenetic trees based on ML and BI inferences revealed the presence of two well-supported clades (Chrysomelini and Doryphorini + Entomoscelini + Gonioctenini), placing the genus *Suinzona* as the sister group of the genus *Potaninia*. This result matched the morphological character of the hind wing. The COI haplotype network of the genus *Suinzona* complex (Fig. 7b) confirms the previous results and shows that the currently known single species is well distinguished as a species. Two independent networks were completely separated without any connection due to the existence of the mutation (62 steps) exceeding the 95% parsimony limits between them.

### Key to *Suinzona borowieci* sp. nov. and related species

1. Hind wings well developed (Fig. 1e); humeral calli present; trichobothria present on anterior angles of pronotum; lateral margins of pronotum distinctly visible from above. China, India, Laos, Myanmar, Thailand and Vietnam........................................................................ *Potaninia assamensis* (Baly, 1879)

– Hind wings reduced (Fig. 1c,d); humeral calli absent; trichobothria absent on anterior angles of pronotum; lateral margins of pronotum not or barely visible from above. China, Korea and Japan........................... 2

2. Aedeagus with apex of flagellum quadrifid (Fig. 1f,g). South Korea, Japan................ 3

– Aedeagus with apex of flagellum varied in shape, but not quadrifid (see Ge et al.^3^ for key to 23 species). China (Sichuan, Yunnan)...................................................... *Suinzona* spp.

3. Larger, body length 5.5–7.0 mm; elytra more densely punctate (Fig. 1a); aedeagus larger and broader (Fig. 2c). South Korea........................................ Suinzona borowieci sp. nov.

– Smaller, body length 4.8–6.0 mm; elytra less densely punctate (Fig. 1d); aedeagus smaller and narrower (Fig. 2e). Japan................................... Suinzona cyrtonoides (Jacoby, 1885)

## Discussion

Members of *Suinzona* exhibit high similarity in their external morphology and colouration, often in the shape of the aedeagus; therefore, the flagellum of the aedeagus was used as the most significant diagnostic character between closely related species. Each species has a specific and complex structure of the flagellum, from bifurcated to concentric circles (see Ge et al.^3^). *S. borowieci* sp. nov. and *S*. *cyrtonoides* comb. nov. show strong similarity in the unique structure of ‘the quadrifurcated tips of the flagellum’, despite the slight difference in the microstructure of the flagellum and the substantial difference in the shape of the aedeagus (Fig. 1f,g). The mature larva of *S*. *borowieci* sp. nov. is very similar to that of *S*. *cyrtonoides* comb. nov. in body shape, colouration and tubercular pattern but differs in the dense setose body and several secondary tubercles between Dae and DLai on the meso- and metathorax. The immature stages and biology of other *Suinzona* species endemic to China are currently unknown. Based on the larval morphology and biology of Korean and Japanese species, the genus *Suinzona* belongs to the generic group *Potaninia* proposed by Kimoto^19^ with the genus *Entomoscelis* Chevrolat and is characterized by the three rows of dorsal tubercles and separated tubercles between Dpe and DLpi on the meso- and metathorax in fourth instar larvae. However, Kimoto^19^ examined only one species, *S*. *cyrtonoides* comb. nov. (as *P. cyrtonoides*), for his classification, and hence the generic group name *Potaninia* needs to be reconsidered. *S. cyrtonoides* comb. nov. and *S*. *borowieci* sp. nov. present a disjunct distribution, with other *Suinzona* species occurring only in Sichuan and Yunnan (Fig. 3a). The former species is endemic to the major Japanese islands (Honshu, Shikoku and Kyushu), and the latter is endemic to South Korea. These two species differ not only in morphology and geographic distribution but also in the range of host plants. *S. cyrtonoides* comb. nov. was recorded feeding only on two *Boehmeria* species from the family Urticaceae (i.e., monophagous), whereas *S*. *borowieci* sp. nov. is associated with four different plant families, namely, Brassicaceae, Lamiaceae, Ranunculaceae and Urticaceae (i.e., polyphagous).

In the phylogenetic analysis of Ge et al.^3^ based on 44 characters of adults and one character of the altitudinal distribution, *P*. *assamensis* and *S*. *cyrtonoides* comb. nov. (as *P*. *cyrtonoides*) formed the *Potaninia* clade, showing a clear divergence from the *Suinzona*-*Taipinus* clade. However, this is probably the result of the misinterpretation of several characters in the character matrix and species description. *S. cyrtonoides* comb. nov. was erroneously described as having a humeral callus and well-developed hind wings. In addition, Kimoto^20^ and Kimoto and Takizawa^11^ wrongly described it as apterous, whereas Suzuki et al.^21^ stated that it has reduced hind wings, a so-called stenopterous, which is in accordance with the type specimens. The flightless leaf beetle *S*. *borowieci* sp. nov. shows high levels of genetic divergence and different host plants at the population level. Each population of this species is small in size and restricted to an extremely narrow habitat area. These effects may lead to allopatric speciation after a long period of time as a result of the increased chance of isolation from other populations. It was demonstrated that the flightless species of carrion beetles retained higher genetic differentiation among the populations than the flying species, and species richness may result from the loss of flight^22^. Unfortunately, several populations of *S*. *borowieci* sp. nov. have declined precipitously over the past few decades, especially in Taebaek and Gunwi. Habitat destruction has occurred due to the creation of new hiking trails or the expansion of agricultural land, which is an important cause of local extinction.

The morphological, molecular and ecological analysis in this study reveals a new species with generic placement from South Korea. The reduced non-functional hind wing of the genus *Suinzona* is the most important generic character that distinguishes it from the genus *Potaninia* and may have promoted population differentiation and species diversification by the reduction in dispersal ability. However, nothing is known about the mitochondrial genome, immature stages and biology of most species (23 species) endemic to southwestern China. Therefore, further studies that include more species are required to obtain a better understanding of the evolution, phylogeny, and biology of the genus *Suinzona* and the closely related genus *Potaninia*.

## Methods

### Museums and private collections

The specimens examined in the study are deposited in the following collections: **BMNH**—Andrzej Warchałowski collection, The National History Museum, London, UK; **EUMJ**—Ehime University Museum, Ehime, Japan; **HCC**—Hee-Wook Cho private collection, Yecheon, South Korea; **HSC**—Haruki Suenaga private collection, Okayama, Japan; **KMNH**—Kitakyushu Museum of Natural History and Human History, Kitakyushu, Japan; **KNAE**—Entomological Collection of Korea National Arboretum, Pocheon, South Korea; **MCZC**—Museum of Comparative Zoology, Harvard University, Cambridge, USA; **NMPC**—Národní Muzeum, Prague, Czech Republic.

### Morphological and biological observations

Field observations were carried out in South Korea in 2004–2011 and 2017–2020 and in Japan in June 2019. The adults of *S. borowieci* sp. nov. collected in the field were maintained in plastic containers (10 cm diameter and 12 cm deep) and laid eggs on the leaves of host plants. All larval specimens used in the study were preserved in 70% ethanol. To examine the morphological characters, some larvae were dissected, cleared in 10% sodium hydroxide solution, rinsed with distilled water, and mounted on slides with glycerine and Swan’s liquid (20 g distilled water, 15 g gum arabic, 60 g chlorhydrate, 3 g glucose, and 2 g glacial acetic acid). Genitalia were dissected from adult specimens softened in plastic containers with wet tissue paper for 12–24 h. The aedeagus was softened in 10% sodium hydroxide solution for 2–6 h and placed in distilled water. The careful insertion of a sharp-pointed thick nose hair and injection of 5% ethanol into the foramen of the aedeagus was repeated until the flagellum and internal sac were fully everted. After washing with absolute ethanol, the genitalia were preserved in a microvial with glycerine and pinned to the specimen. Descriptions and illustrations were prepared using Nikon SMZ800 and Nikon Eclipse E600 microscopes, each equipped with a drawing tube. Photographs were taken by a Nikon D850 digital camera attached to a Leica M165C microscope and were edited in Helicon Focus 7.6.4 and Adobe Photoshop 2020. Line drawings were made in Adobe Photoshop 2020 with a Wacom Intuos4 graphics tablet from photographs. In the larval description, the letters L, S, and M after Arabic numerals within parentheses signify long, short, and minute setae on the tubercle, respectively. The terminology used on the larval tubercle is as described by Kimoto^19^.

### DNA extraction and sequencing

Total genomic DNA was extracted from three species of *Suinzona* and *Potaninia* samples using a DNeasy Blood & Tissue Kit (Qiagen Co., Germany) following the manufacturer’s protocol. PCR conditions and primers for the mitochondrial COI gene (LCO1490 + eCOI—2H) following Oba et al.^23^. The obtained sequences were deposited in GenBank (Supplementary Table S1). Mitochondrial genomes were sequenced using shotgun sequencing on the HiSeq 2000 platform using libraries with an insert size of 200 bp and paired-end sequencing of 100 bp. Assembly and annotation of genes were performed as described by Nie et al.^24^, using *Entomoscelis adonis* (GenBank: KX943493) as a reference. The obtained mitochondrial genome sequences were deposited in GenBank (Supplementary Table S1).

### Phylogenetic analyses

All the PCG and rRNA genes were aligned individually using Muscle 3.8.425^25^. The aligned data from each mitochondrial gene were concatenated with Geneious V.2021.0.3^26^. Phylogenetic inferences were performed using MrBayes 3.2.7^27^ and raxmlGUI 2.0^28^. For the BI and ML analyses, the best-fit models of nucleotide substitution and partition schemes were selected using PartitionFinder 2^29^. BI analyses were run for 20 million generations initiated with program-generated trees, two independent runs of four Markov chain Monte Carlo (MCMC) chains and sampling every 100 generations. The first 25% of trees were discarded as burn-in and then visualized using FigTree v1.4.4^30^. For the ML analysis, the GTR-CAT model was chosen for the bootstrapping phase, conducted with initial tree searches, followed by 10,000 ultrafast bootstrap replicates. Sequences of the COI gene were also constructed in unrooted parsimonious networks using TCS 1.21^31^ with a connection limit of 80%.

### Nomenclatural acts

The electronic edition of this article meets the requirements of the amended International Code of Zoological Nomenclature, and hence, the new names contained herein are available under that Code from the electronic edition of this article. This published work and the nomenclatural acts it contains have been registered in ZooBank, the online registration system for the ICZN. The ZooBank Life Science Identifiers (LSIDs) can be resolved and the associated information can be viewed through any standard web browser by appending the LSID to the prefix “http://zoobank.org/”. The LSID for this publication is urn:lsid:zoobank.org:act:5DC70067-DB70-4DE3-A4E6-AD287247D19F. The electronic edition of this paper was published in a journal with an ISSN, and it has been archived and is available from PubMed Central.

## Supplementary Information

Supplementary Information

## Data Availability

The type series of the new species is deposited in the Národní Muzeum, Prague, Czech Republic [NMPC] and H.-W. Cho’s private collection [HCC]. The molecular sequences obtained in this study are available in GenBank. The sequence accession numbers and collection locality for each specimen are presented in Supplementary Table S1.
